# Timing of First Antenatal Care (ANC) and Inequalities in Early Initiation of ANC in Nepal

**DOI:** 10.3389/fpubh.2017.00242

**Published:** 2017-09-11

**Authors:** Yuba Raj Paudel, Trishna Jha, Suresh Mehata

**Affiliations:** ^1^FAIRMED Nepal, Lalitpur, Nepal; ^2^National Open College, Lalitpur, Nepal; ^3^Nepal Public Health Foundation, Kathmandu, Nepal

**Keywords:** antenatal care, early initiation, inequality, Nepal, timing

## Abstract

**Background:**

The provision and uptake of quality and timely antenatal care (ANC) is an essential element of efforts to improve health outcomes for women and newborn babies. Antenatal consultations assist in early identification and treatment of complications during pregnancy. This study aimed to provide an information on distribution and inequalities in early initiation of ANC in Nepal.

**Methods:**

The distribution and inequalities in the early initiation of ANC were examined using Nepal Demographic and Health Surveys 2011. Bivariate and multivariate logistic regression was used to assess inequalities.

**Findings:**

Overall, 70% of the women had started their first ANC at 4 month or earlier. Among participants who had never attended school, just more than half (52%) received first ANC at 4 months or earlier, while majority of participants (97%) who had received higher education received first ANC at recommended time. Similarly, 89% of those from richest quintile and 48% of those from poorest quintile received first ANC at recommended time. In adjusted analysis, women from richest wealth quintile were significantly more likely to initiate ANC early (AOR: 3.74, 95% CI: 2.31–6.05) compared to the poorest. Similarly, women with higher level education were significantly more likely (AOR: 11.40, 95% CI: 5.05–25.73) to initiate ANC early compared to women who had never attended school. A significantly lower odds of early ANC take up was observed among madhesi other caste (AOR: 0.56, 95% CI: 0.35–0.90) compared to brahmin/chhetri women. Women whose pregnancy was unwanted were significantly less likely to attend first ANC at 4 months or early (AOR: 0.73, 95% CI: 0.58–0.93) in comparison to women whose pregnancy was wanted.

**Conclusion:**

The differences in the recommended timing of initiation of ANC were evident among women with different educational, economic levels, and caste/ethnic groups. Rural women were less likely to have checkups as per guidelines. The findings suggest to a need of interventions to raise female education and improve economic status of households. Targeted interventions suitable to local context and culture are equally important. Increasing access to family planning methods and reduction of unwanted pregnancy can promote early ANC take up.

## Introduction

Antenatal care (ANC) is an important entry point for pregnant women to receive health promotion and preventive information and services including iron supplementation, deworming tablet, tetanus injection, and malaria prophylaxis (1, 2). Similarly, early entry to ANC helps health workers to provide timely information and services according to the gestational age and health condition. On the contrary, mothers who attend ANC late miss the opportunity to receive health information and interventions such as early detection of HIV, malaria, and anemia prophylaxis, and prevention or management of complications (3).

Several studies have shown that women who started ANC attendance early and attended frequently were more likely to be assisted during childbirth by a skilled attendance compared to those who initiated ANC late and attended only few visits (4, 5) Late or no ANC has been reported to be associated with poor outcomes for mother and fetus such as premature birth, still birth (6), low birth weight (5), and increased complications during pregnancy and childbirth (7).

Evidence suggests not knowing the right gestation age at which to start the first ANC visit was most common reason for late attendance (8, 9). Studies have shown cultural factors (10), quality of care (4), economic factors (11), women’s educational status (11), poor knowledge on timing of first ANC visit (9), and access to ANC to be determining when a woman starts her ANC checkup. The findings suggest adequate provision of information and counseling concerning early attendance of ANC could have an important role (12). Community-based intervention involving community volunteers have been found to be effective to promote early and adequate ANC visit (4).

Government of Nepal (GoN) recommends focused antenatal visits at fourth, sixth, eighth, and ninth months of pregnancy (2). In Nepal, ANC services are provided as a component of primary health-care services for pregnant women. ANC services are available from all public health facilities in Nepal including from community level primary health-care outreach clinics (PHC-ORC) (2). Similarly, female community health volunteers (FCHVs) carry-out awareness raising activities and iron, folic acid distribution at the community level. Furthermore, GoN has initiated Aama program (free maternity care and transport incentives to promote four ANC visit and institutional delivery) since 2009 (13).

Although trend of ANC utilization is increasing in Nepal, only half of the pregnant women attend four recommended antenatal visits. However, there is a paucity of data on facilitators and barriers to early initiation of ANC visits in Nepal. This study will provide important information to health policy makers on what influences timing of first ANC visit in Nepal and inequalities.

## Materials and Methods

### Data Source and Sampling Strategy

This study used data from the NDHS 2011, and details of the NDHS methodology, sampling, and detail questionnaires are available in the report (14). Briefly, the NDHS 2011 used two-stage stratified cluster sampling to select a representative sample of households. The primary objective was to provide national estimates of major indicators related to status of access and utilization of reproductive health care in Nepal. A total of 11,353 households were selected from 289 primary sampling units (194 rural and 95 urban) using probability proportionate to size. An approximate response rate of 99% was reported for households that were occupied. This analysis is based on 4,148 women who had a live birth during 5 years preceding the survey.

### Variables Used for Analysis

The dependent variable in this analysis was early initiation of ANC. Early initiation of ANC was defined as attending first ANC at 4 months of pregnancy or earlier. This variable was coded “1” if women reported attending at 4 months or earlier and “0” if she did not attend ANC or attended after 4 months. Six explanatory variables studied include: age of the mother, place and ecological region of residence, educational status of women, wealth quintiles, ethnicity, development region and pregnancy wantedness.

### Data Analysis

To provide population level estimates, reported values were weighted by sample weights. Bivariate and multivariate logistic regression analysis was used to measure the inequalities between the factors and outcome variables using complex survey design, considering clusters, and stratification by urban/rural location. A *P* value of less than 0.05 was considered to be statistically significant.

### Ethics

Since the current study was based on data from Nepal Demographic Health Survey 2011, no separate ethical approval was sought. Ethical approvals for NDHS 2011 were obtained from the Nepal Health Research Council ethical review committee.

## Results

Table 1 shows the timing of the first ANC visit. The table shows that the peak month for the first ANC visit is the third month of pregnancy: nearly one-third (29%) of women attended their first checkup in that month. Over two-third (70%) had their first checkup within the first 4 months.

**Table 1:** Month of first antenatal care (ANC) visit.

	*N*	%
First month	321	7.73
Second month	552	13.30
Third month	1,187	28.62
Fourth month	716	17.27
Fifth month	365	8.80
Sixth month	241	5.81
Seventh month	67	1.61
Eighth month	47	1.14
Ninth month	22	0.54
No ANC	629	15.15
Total	4,148	100.00

Those residing in Terai districts (71%) were more likely to have received their first ANC checkup in the first four months than those in mountain (58%) (Table 2). The likelihood of having had an ANC checkup in the first 4 months increased with increasing levels of education, from 52% for those who had never attended school to 97% for those with higher level education. People from madhesi other caste (57%) were least likely to have had an ANC checkup in the first 4 months. Uptake by Dalits (61%) and Muslims (62%) was also low. The likelihood of having had an ANC checkup in the first 4 months increased with increasing levels of economic status, from 48% for those who were poorest quintile to 89% for those were highest quintile.

**Table 2:** Distribution of time of first antenatal care—at 4 months or earlier.

	4 months or earlier		Other		Total (*N*)
	*N*	%	*N*	%	
**Age group**					
<20	235	70.6	98	29.4	333
20–34	2,296	69.4	1,013	30.6	3,309
35–49	245	48.4	261	51.6	507
**Region**					
Mountain	178	58.1	128	41.9	306
Hill	1,066	63.9	602	36.1	1,669
Terai	1,533	70.5	642	29.5	2,174
**Place of residence**					
Urban	330	79.0	88	21.0	418
Rural	2,446	65.6	1,285	34.4	3,730
**Highest educational level**					
No education	944	51.8	878	48.2	1,822
Primary	555	66.5	280	33.5	835
Secondary	1,024	83.3	205	16.7	1,229
Higher	254	96.5	9	3.5	263
**Wealth index**					
Poorest	474	48.4	505	51.6	979
Poorer	545	60.6	355	39.4	899
Middle	600	68.8	272	31.2	873
Richer	578	77.2	170	22.8	748
Richest	580	89.3	70	10.7	649
**Caste/ethnicity**					
Brahmin/chhetri	980	76.4	303	23.6	1,283
Madhesi other caste	236	57.1	177	42.9	414
Dalit	416	61.0	266	39.0	683
Newar	111	87.2	16	12.8	127
Janajati	880	63.0	516	37.0	1,396
Muslims	145	61.5	91	38.5	236
Others	8	77.8	2	22.2	10
**Development region**					
Eastern	712	71.3	287	28.7	999
Central	808	62.5	485	37.5	1,293
Western	543	66.4	275	33.6	818
Mid-western	392	65.5	206	34.5	598
Far-western	321	72.9	119	27.1	440
**Pregnancy wantedness**					
Wanted then	2,085	69.1	932	30.9	3,017
Wanted later	372	74.5	127	25.5	499
No more wanted	319	50.5	313	49.5	632
**Total**	**2,776**	**66.9**	**1,372**	**33.1**	**4,148**

### Inequalities in Early Initiation of ANC

Current analysis showed notable inequalities in early initiation of ANC in Nepal (Table 3). Women from the richest wealth quintile were almost four times more likely to initiate ANC at 4 month or earlier (AOR: 3.74, 95% CI: 2.31–6.05). Compared to women had had no education, more than 11 times more like to initiate ANC among women educated at higher level (AOR: 11.40, 95% CI: 5.05–25.73). Compared to brahmin/chhetri, the significantly lower odds was observed among madhesi other caste (AOR: 0.56, 95% CI: 0.35–0.90). Furthermore, women who reside in far-western region were almost twice likelihood of earlier initiation of ANC compared to women resides in eastern regions (AOR: 1.67; 95% CI: 1.13–2.46).

**Table 3:** Inequalities in the early initiation first antenatal care: findings from bivariate and multivariate logistic regression.

	Univariate				Multivariate			
	Crude OR	*P*	95% CI		Adjusted OR	*P*	95% CI	
**Age group**								
<20	1.00				1.00			
20–34	0.68	0.01	0.50	0.92	1.00	0.99	0.70	1.44
35–49	0.40	<0.001	0.28	0.57	0.80	0.33	0.51	1.25
**Ecological zone**								
Mountain	1.00				1.00			
Hill	1.41	0.02	1.05	1.89	1.10	0.59	0.78	1.55
Terai	1.66	<0.001	1.23	2.24	1.45	0.05	1.00	2.09
**Place of residence**								
Urban	1.00				1.00			
Rural	0.52	<0.001	0.40	0.68	1.09	0.55	0.82	1.46
**Education level**								
No education	1.00				1.00			
Primary	1.80	<0.001	1.50	2.16	1.62	<0.001	1.26	2.08
Secondary	3.93	<0.001	3.24	4.78	2.94	<0.001	2.19	3.94
Higher	10.48	<0.001	7.14	15.38	11.40	<0.001	5.05	25.73
**Wealth quintile**								
Poorest	1.00				1.00			
Poorer	1.65	<0.001	1.36	2.01	1.49	<0.001	1.16	1.91
Middle	2.18	<0.001	1.66	2.86	1.86	<0.001	1.37	2.53
Richer	3.11	<0.001	2.38	4.06	2.25	<0.001	1.61	3.15
Richest	6.66	<0.001	5.00	8.88	3.74	<0.001	2.31	6.05
**Caste/ethnicity**								
Brahmin/chhetri	1.00				1.00			
Madhesi other caste	0.48	<0.001	0.32	0.71	0.56	0.02	0.35	0.90
Dalit	0.50	<0.001	0.39	0.64	0.93	0.67	0.68	1.28
Newar	2.38	<0.001	1.33	4.26	1.55	0.17	0.83	2.91
Janajati	0.66	<0.001	0.50	0.86	0.79	0.09	0.60	1.04
Muslims	0.46	<0.001	0.34	0.62	0.87	0.58	0.53	1.42
Others	1.15	0.82	0.35	3.79	0.62	0.66	0.07	5.16
**Development region**								
Eastern	1.00				1.00			
Central	0.70	0.03	0.50	0.96	0.78	0.16	0.56	1.10
Western	0.92	0.63	0.64	1.31	0.76	0.12	0.53	1.08
Mid-western	0.76	0.14	0.53	1.09	1.21	0.37	0.80	1.83
Far-western	0.88	0.47	0.63	1.24	1.67	0.01	1.13	2.46
**Wanted last child**								
Wanted then	1.00				1.00			
Wanted later	1.31	0.06	0.98	1.75	0.93	0.64	0.70	1.25
Wanted no more	0.46	<0.001	0.37	0.57	0.73	0.01	0.58	0.93

## Discussion

Importance of early and adequate ANC for health and well-being of mothers and children remains undisputed. However, various individual, family level, and contextual factors influence women’s utilization of ANC (11). Current study focused on timing of first ANC and inequalities in early initiation of ANC drawing data from NDHS 2011. This study did not study frequency and quality/content of ANC since a previous study has investigated this issue (15).

Just two-third women (70%) received first ANC at 4 months or earlier. In other words, nearly one-third pregnant women in Nepal still miss the opportunity to early receive various preventive and health promotion services and detection of pregnancy related complications. Various cultural factors might act as barriers for women to initiate early ANC services. Women in some culture may want to keep their pregnancy secret to avoid harm from other people or spirits (4). Pregnant women might face resistance within the home to go for ANC since a qualitative study conducted in Nepal showed a significant role of mother-in-laws in pregnant women’s utilization of ANC (10). In this context, targeting mother-in-laws and husbands in health promotion and educational interventions could be a promising strategy in Nepal to promote early ANC utilization (10). A randomized controlled trial in conducted in urban area of Nepal showed a positive effect of couple education on maternal health-care utilization (16).

In the current analysis, the household wealth quintile showed a significant relationship with early initiation of ANC. A multi-country comparative study also has revealed non-use of maternal health care including ANC to be concentrated among poor women (17). Women from richer households may have more autonomy, better education, and better skill and confidence to deal with service providers compared to poorer women (18). In addition, hidden costs such as cost of transportation, cost of diagnostic and opportunity cost due to loss of wage could also be acting as barriers for timely initiation of ANC by women from poor households. Furthermore, poorer women are less likely to get permission from husband and family members to visit health facility for ANC checkup due to agricultural workload or other commitments. Similarly, experience of mistreatment in previous visits by poorer women from health-care providers could be associated with hesitation to start ANC at early stage (18, 19). Hence, even in places where services are readily available, impoverishment can lead to delayed receipt of ANC care.

Women from Terai region of Nepal were more likely to attend early ANC. Factors such as easy access to transportation; easier access to health facilities could be facilitating factors for women to attend ANC early in Terai region of Nepal. Less distance to nearest health post from home could be a facilitating factor for early ANC visit since a study conducted in western Nepal showed positive association of ANC visit with less distance (20). Nepal’s mountain and hill region have difficult topography and women have to walk for hours if not days to reach to a health facility. Although ANC is provided through PHC-ORC clinics—community-based outreach clinics conducted once a month—these clinics are mostly non-functional and have very poor infrastructures often running in open space.

The educational level of women showed an apparent dose response relationship with early initiation of ANC. Educated mothers might be more likely to be well informed about benefits of ANC checkup. This finding is in line with earlier studies from Bangladesh (21) and Africa (22). Illiterate women might have limited understanding of ANC services and their importance to ensure safe delivery (23). Stephenson and Tsui revealed women in Uttar Pradesh, India thought pregnancy as a natural process that only warranted ANC when problems arose (11). Since health care in Nepal is also mostly considered to be curative, pregnant women in Nepal might perceive that ANC is needed only when complications arise.

Several studies report late or inadequate utilization of ANC by adolescent mothers (22, 24–27); however;, current study did not find an association of age with late initiation of ANC. Although, women above the age of 35 were more likely to initiate ANC late compared to adolescent mothers, the difference was not significant in multivariate analysis. Parity could be a confounder since Matthews et al found that higher order births were associated with late or inadequate ANC in Karnataka, India (26). Researchers suggest that higher parity women may not use adequate ANC due to their increased confidence from experience of previous pregnancy and childbirth, or due to time and resource constrains caused by a larger family (28). A study conducted in Tanzania also showed that primiparous women started ANC 3 weeks earlier in comparison to multiparous women (4). Late initiation of ANC by older age mothers could also be due to poorer quality of care in health facility during previous pregnancy and childbirth (29).

Similar to this study, inequalities by caste/ethnicity in utilization of ANC has been documented elsewhere and in Nepal (30, 31). A study conducted in Kenya showed that disadvantaged ethnicities were more likely to initiate ANC late compared to advantaged ethnicities (31). Kurdish women were less likely to use ANC services in Turkey (32). In our study, women from madhesi other castes initiated ANC much later than other castes despite hailing from geographically accessible plain area of Nepal. Madhesi other castes are middle caste living in Nepal’s southern plain region (Terai). They are economically strong but women’s literacy, women empowerment, and health indicators are very poor. Gender and social inequalities are much higher in this caste (33). This finding signals to the sociocultural barriers prevalent in Nepal’s Terai region (30).

Consistent with several studies (31, 34, 35), current study found a link between having experienced unwanted pregnancy and late initiation of ANC. It has been reported that women want to conceal their unintended pregnancy by delaying or avoiding ANC visit (22). Wanting to terminate pregnancy could also be another reason for delaying ANC among women experiencing unintended pregnancy. Given the lower utilization of family planning by young, rural (36), and less educated couples in Nepal (37), increasing access to quality FP service to these groups needs to be a priority. Furthermore, stronger negative association of early initiation of ANC with unwanted pregnancy compared to mistimed pregnancy suggest to a need of increasing access to limiting methods of family planning.

The findings from this study present several policy implications. First, information on timing of first ANC and benefits of early initiation need to be delivered through media, and health workers including FCHVs. A clear and consistent message regarding pregnancy risks and importance of ANC to protect health of mother and child need to be disseminated. Women’s education with emphasis on increasing health knowledge by including sexual and reproductive health topics in educational curriculum might be game changers on maternal health-care utilization. Strategies need to be developed to reach to poorer groups or more research is needed to design approaches to reach them. Different contexts (ecological regions) may require different interventions to reduce inequalities in ANC utilization. A provision of home-based care (38) might be effective to promote timely ANC among hard to reach populations. Increasing access to family planning among unreached population will reduce the likelihood of unintended pregnancy and will eventually increase the likelihood of early initiation of first ANC visit. In the light of findings from this study and previous findings (39), it seems imperative that the strategy to incentivize four ANC and institutional delivery need improvement with better targeting.

## Conclusion

Adequate and timely ANC is vital in achieving good maternal and child health outcomes in developing countries such as Nepal (40). Our findings suggest that interventions to improve women’s educational status have independent effect on early initiation of ANC. Health interventions through information, education, and communication programs adapted to local contexts can promote timely ANC checkups. Removing financial barriers among the poor from the poor region also could improve early initiation of ANC. Access to family planning services, especially limiting methods need to be increased to reduce unwanted pregnancy eventually to promote early ANC.

## Author Contributions

SM and YP had the concept for the paper, prepared methodology, and conducted data analysis. SM, YP, and TJ conducted literature review and prepared first draft. All the authors reviewed and agreed on the first draft of the article.

## Conflict of Interest Statement

The authors declare that the research was conducted in the absence of any commercial or financial relationships that could be construed as a potential conflict of interest.

## References

[1] Wehby GL Murray JC Castilla EE Lopez-Camelo JS Ohsfeldt RL. Prenatal care effectiveness and utilization in Brazil. Health Policy Plan (2009) 24:175–88. 10.1093/heapol/czp005 19282483 PMC2708921

[2] Department of Health Services. Annual Report – 2012/13. Kathamandu (2014).

[3] Belayneh T Adefris M Andargie G. Previous early antenatal service utilization improves timely booking: cross-sectional study at university of Gondar hospital, northwest Ethiopia. J Pregnancy (2014) 2014:132494. 10.1155/2014/132494 25101176 PMC4102065

[4] Gross K Alba S Glass TR Schellenberg JA Obrist B. Timing of antenatal care for adolescent and adult pregnant women in south-eastern Tanzania. BMC Pregnancy Childbirth (2012) 12(1):16. 10.1186/1471-2393-12-16 22436344 PMC3384460

[5] Beeckman K Louckx F Masuy-Stroobant G Downe S Putman K. The development and application of a new tool to assess the adequacy of the content and timing of antenatal care. BMC Health Serv Res (2011) 11(1):213. 10.1186/1472-6963-11-213 21896201 PMC3176177

[6] Beauclair R Petro G Myer L. The association between timing of initiation of antenatal care and stillbirths: a retrospective cohort study of pregnant women in Cape Town, South Africa. BMC Pregnancy Childbirth (2014) 14(1):204. 10.1186/1471-2393-14-204 24923284 PMC4062506

[7] Heaman M Newburn-Cook C Green C Elliott L Helewa M. Inadequate prenatal care and its association with adverse pregnancy outcomes: a comparison of indices. BMC Pregnancy Childbirth (2008) 8(1):15. 10.1186/1471-2393-8-15 18452623 PMC2386440

[8] Kisuule I Kaye DK Najjuka F Ssematimba SK Arinda A Nakitende G Timing and reasons for coming late for the first antenatal care visit by pregnant women at Mulago hospital, Kampala Uganda. BMC Pregnancy Childbirth (2013) 13:121 10.1186/1471-2393-13-121 23706142 PMC3665546

[9] Ndidi EP Oseremen IG. Reasons given by pregnant women for late initiation of antenatal care in the Niger Delta, Nigeria. Ghana Med J (2010) 44(2):47–51. 10.4314/gmj.v44i2.68883 21327003 PMC2994152

[10] Simkhada B Porter MA Van Teijlingen ER. The role of mothers-in-law in antenatal care decision-making in Nepal: a qualitative study. BMC Pregnancy Childbirth (2010) 10(1):34. 10.1186/1471-2393-10-34 20594340 PMC2910658

[11] Stephenson R Tsui AO. Contextual influences on reproductive health service use in Uttar Pradesh, India. Stud Fam Plann (2002) 33(4):309–20. 10.1111/j.1728-4465.2002.00309.x 12561780

[12] Alderliesten ME Vrijkotte TG van der Wal MF Bonselb GJ. Late start of antenatal care among ethnic minorities in a large cohort of pregnant women. BJOG (2007) 114:1232–9. 10.1111/j.1471-0528.2007.01438.x 17655734

[13] Family Health Division. Aama Program Guideline, Second Revision 2069. Kathmandu: Family Health Division, DoHS (2012).

[14] Ministry of Health and Population (MOHP) [Nepal], New ERA, ICF International Inc. Nepal Demographic and Health Survey 2011. Kathmandu, Nepal; Calverton, Maryland: Ministry of Health and Population, New ERA; ICF International (2012).

[15] Joshi C Torvaldsen S Hodgson R Hayen A. Factors associated with the use and quality of antenatal care in Nepal: a population-based study using the demographic and health survey data. BMC Pregnancy Childbirth (2014) 14(1):94. 10.1186/1471-2393-14-94 24589139 PMC3943993

[16] Mullany BC Becker S Hindin M The impact of including husbands in antenatal health education services on maternal health practices in urban Nepal: results from a randomized controlled trial. Health Educ Res (2006) 22(2):166–76. 10.1093/her/cyl060 16855015

[17] Houweling TA Ronsmans C Campbell OM Kunst AE. Huge poor-rich inequalities in maternity care: an international comparative study of maternity and child care in developing countries. Bull World Health Organ (2007) 85(10):745–54. 10.2471/BLT.06.038588 18038055 PMC2636501

[18] Cleland JG Van Ginneken JK. Maternal education and child survival in developing countries: the search for pathways of influence. Soc Sci Med (1988) 27(12):1357–68. 10.1016/0277-9536(88)90201-8 3070762

[19] Moyer CA Adongo PB Aborigo RA Hodgson A Engmann CM ‘They treat you like you are not a human being’: maltreatment during labour and delivery in rural northern Ghana. Midwifery (2014) 30(2):262–8. 10.1016/j.midw.2013.05.006 23790959

[20] Choulagai B Onta S Subedi N Mehata S Bhandari GP Poudyal A Barriers to using skilled birth attendants’ services in mid-and far-western Nepal: a cross-sectional study. BMC Int Health Hum Rights (2013) 13(1):49 10.1186/1472-698X-13-49 24365039 PMC3878020

[21] Kamal SM Hassan CH Islam MN Factors associated with the timing of antenatal care seeking in Bangladesh. Asia Pac J Public Health (2013) 27:NP1467–80. 10.1177/1010539513485786 24097925

[22] Magadi MA Agwanda AO Obare FO A comparative analysis of the use of maternal health services between teenagers and older mothers in sub-Saharan Africa: evidence from demographic and health surveys (DHS). Soc Sci Med (2007) 64(6):1311–25. 10.1016/j.socscimed.2006.11.004 17174017

[23] Andrew EVW Pell C Angwin A Auwun A Daniels J Mueller I Factors affecting attendance at and timing of formal antenatal care: results from a qualitative study in Madang, Papua New Guinea. PLoS One (2014) 9(5):e93025. 10.1371/journal.pone.0093025 24842484 PMC4026245

[24] Bearinger LH Sieving RE Ferguson J Sharma V. Global perspectives on the sexual and reproductive health of adolescents: patterns, prevention, and potential. Lancet (2007) 369(9568):1220–31. 10.1016/S0140-6736(07)60367-5 17416266

[25] Van Eijk AM Bles HM Odhiambo F Ayisi JG Blokland IE Rosen DH Use of antenatal services and delivery care among women in rural western Kenya: a community based survey. Reprod Health (2006) 3(1):2. 10.1186/1742-4755-3-2 16597344 PMC1459114

[26] Matthews Z Mahendra S Kilaru A Ganapathy S Antenatal care, care-seeking and morbidity in rural Karnataka, India: results of a prospective study. Asia Pac Popul J (2001) 16(2):11–28.

[27] Miles-Doan R Brewster KL. The impact of type of employment on women’s use of prenatal-care services and family planning in urban Cebu, the Philippines. Stud Fam Plann (1998) 29:69–78. 10.2307/172182 9561670

[28] Navaneetham K Dharmalingam A. Utilization of maternal health care services in Southern India. Soc Sci Med (2002) 55(10):1849–69. 10.1016/S0277-9536(01)00313-6 12383469

[29] Trinh LTT Dibley MJ Byles J. Determinants of antenatal care utilization in three rural areas of Vietnam. Public Health Nurs (2007) 24(4):300–10. 10.1111/j.1525-1446.2007.00638.x 17553019

[30] Deo KK Paudel YR Khatri RB Bhaskar RK Paudel R Mehata S Barriers to utilization of antenatal care services in Eastern Nepal. Front Public Health (2015) 3:197. 10.3389/fpubh.2015.00197 26322302 PMC4536371

[31] Magadi MA Madise NJ Rodrigues RN. Frequency and timing of antenatal care in Kenya: explaining the variations between women of different communities. Soc Sci Med (2000) 51(4):551–61. 10.1016/S0277-9536(99)00495-5 10868670

[32] Celik Y Hotchkiss DR. The socio-economic determinants of maternal health care utilization in Turkey. Soc Sci Med (2000) 50(12):1797–806. 10.1016/S0277-9536(99)00418-9 10798333

[33] Bennett L Dahal DR Govindasamy P Caste Ethnic and Regional Identity in Nepal: Further Analysis of the 2006 Nepal Demographic and Health Survey. Calverton: Macro International Inc. (2008).

[34] Paredes I Hidalgo L Chedraui P Palma J Eugenio J. Factors associated with inadequate prenatal care in Ecuadorian women. Int J Gynecol Obstet (2005) 88(2):168–72. 10.1016/j.ijgo.2004.09.024 15694103

[35] Erci B. Barriers to utilization of prenatal care services in Turkey. J Nurs Scholarsh (2003) 35(3):269–73. 10.1111/j.1547-5069.2003.00269.x 14562496

[36] Mehata S Paudel YR Dotel BR Singh DR Poudel P Barnett S. Inequalities in the use of family planning in rural Nepal. Biomed Res Int (2014) 2014:636439. 10.1155/2014/636439 25405205 PMC4163397

[37] Adhikari R Soonthorndhada K Prasartkul P. Correlates of unintended pregnancy among currently pregnant married women in Nepal. BMC Int Health Hum Rights (2009) 9(1):17. 10.1186/1472-698X-9-17 19671161 PMC2731722

[38] Baqui AH El-Arifeen S Darmstadt GL Ahmed S Williams EK Seraji HR Effect of community-based newborn-care intervention package implemented through two service-delivery strategies in Sylhet district, Bangladesh: a cluster-randomised controlled trial. Lancet (2008) 371(9628):1936–44. 10.1016/S0140-6736(08)60835-1 18539225

[39] Ensor T Bhatt H Tiwari S Incentivizing universal safe delivery in Nepal: 10 years of experience. Health Policy Plan (2017). 10.1093/heapol/czx070 28591799

[40] Dražančić A Antenatal care in developing countries. What should be done? J Perinat Med (2001) 29(3):188–98. 10.1515/JPM.2001.028 11447923

